# Finding the genes for fragile bones

**DOI:** 10.7554/eLife.85161

**Published:** 2022-12-23

**Authors:** Erika Kague

**Affiliations:** 1 School of Physiology, Pharmacology and Neuroscience, University of Bristol Bristol United Kingdom

**Keywords:** TWAS, GWAS, osteoporosis, eQTL, Human, Mouse

## Abstract

Combining transcriptomic data with the analysis of large genome-wide association studies helps identify genes that are likely important for regulating bone mineral density.

**Related research article** Al-Barghouthi BM, Rosenow WT, Du K-P, Heo J, Maynard R, Mesner L, Calabrese G, Nakasone A, Senwar B, Gerstenfeld L, Larner J, Ferguson V, Ackert-Bicknell C, Morgan E, Brautigan D, Farber CR. 2022. Transcriptome-wide association study and eQTL colocalization identify potentially causal genes responsible for human bone mineral density GWAS associations. *eLife* **11**:e77285. doi: 10.7554/eLife.77285.

Bone fractures are painful and debilitating, particularly as we get older. However, some people are more susceptible than others to breaking bones as they age. Osteoporosis is the most prevalent bone condition of the ageing population (Salari et al., 2021). It is characterized by low bone mass and the deterioration of bone microarchitecture, leading to porous bones that are more likely to break (Trajanoska et al., 2018). But what are the causes of osteoporosis?

Studies involving twins and families have taught us that osteoporosis is heritable (Ralston and Uitterlinden, 2010), which means that genetics play a vital role in determining whether someone is at risk of developing osteoporosis. Learning which genes cause osteoporosis could teach us how the condition develops, opening therapeutic avenues to treat it.

This rationale has inspired researchers to perform genome-wide association studies (GWAS) to identify regions of the genome associated with bone mineral density, which is the trait that is the best indicator of fracture risk (Timpson et al., 2018). It has been 15 years since the first bone mineral density GWAS (Kague et al., 2022). The largest of these studies, published in 2019, involved around 425,000 participants and successfully identified over 500 genomic regions associated with bone mineral density of the heel (Morris et al., 2019).

However, interpreting the associations captured with GWAS can be challenging, because the loci that are identified can contain several genes, reside in non-coding regions near genes, or contain no genes at all. Therefore, despite the remarkable progress made by bone mineral density GWAS, the field needs to bridge a considerable gap that lies between correlation and causation. Filling this gap is essential to understand the underlying biology of osteoporosis and find potential therapeutics for the condition.

Now, in eLife, Charles R Farber and colleagues from the University of Virginia, the University of Colorado and Boston University – including Basel Maher AI-Barghouthi as first author – report on a systematic way to narrow down GWAS signals and shed light on the genes that are likely responsible for variations in human bone mineral density (Al-Barghouthi et al., 2022). To do this, the group leveraged transcriptomic data and computational approaches to systematically screen published bone mineral density GWAS (Figure 1). They identified 512 genes that could be regulators of bone mineral density, including the gene PPP6R3, which had not been associated with bone before.

**Figure 1.**
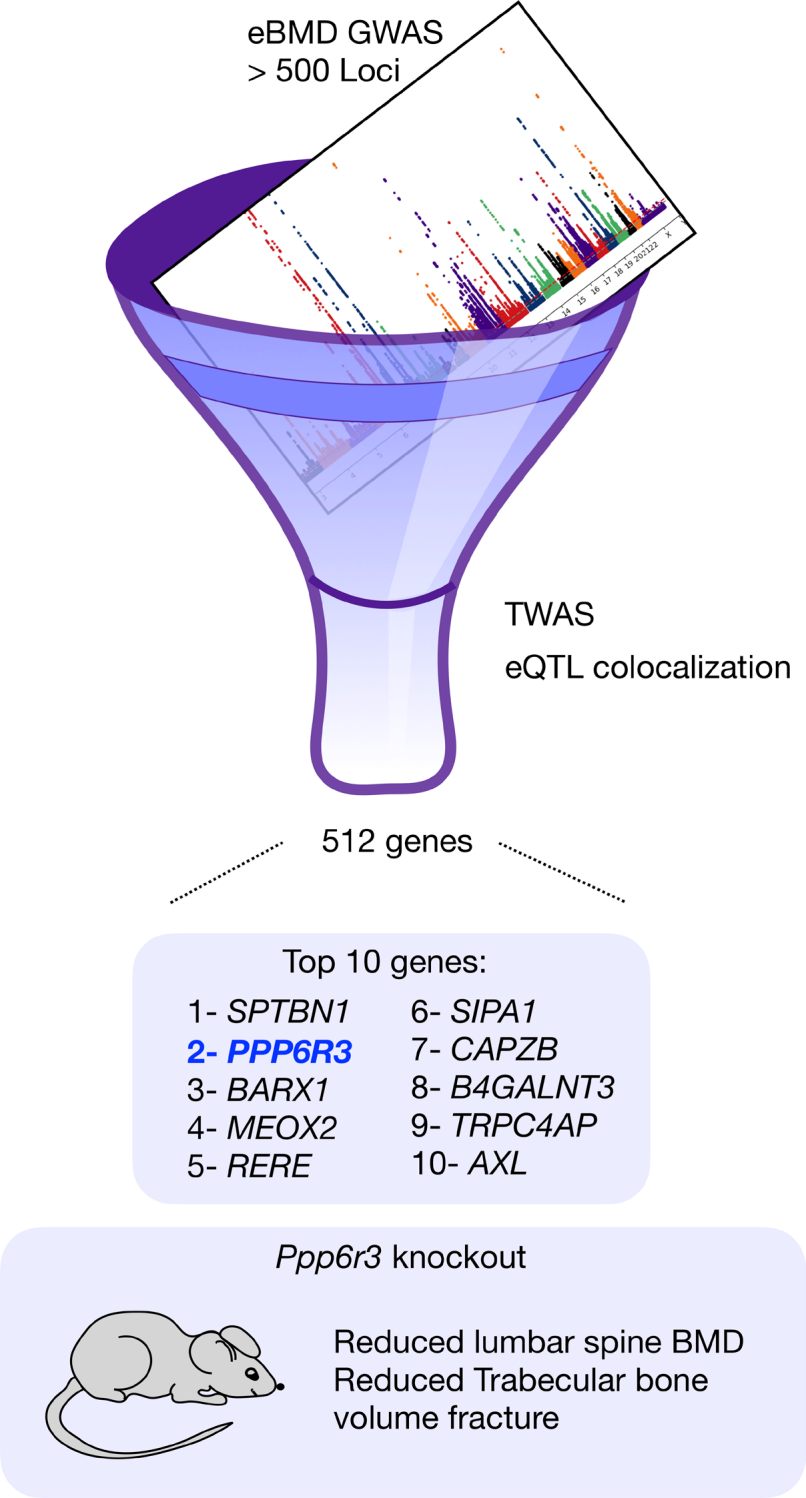
Combining two computational approaches identifies genes that could cause osteoporosis. The results from a genome-wide association study (GWAS) that identified over 500 genomic regions that were associated with low bone mineral density in the heel were filtered using a combination of two techniques – transcriptome-wide association studies and expression quantitative trait loci (eQTL) colocalization – and this resulted in the identification of 512 genes that could be causing osteoporosis. The 10 most significant genes are shown. The gene with the second highest score, PPP6R3, had not previously been linked to bone. Al-Barghouthi et al. went on to show that mice in which this gene had been knocked out had reduced bone mineral density in their vertebrae.

Al-Barghouthi et al. used data from the Genotype-Tissue Expression (GTEx) project, which is an ongoing public resource containing gene expression data from 54 healthy tissue sites across nearly 1000 individuals whose genomes have been sequenced (GTEx Consortium, 2013). GTEx data allows researchers to infer how DNA variations in the proximity of a gene can influence fluctuations in gene expression, what is known as expression quantitative trait loci (eQTL). The team used this information to determine which of the genomic regions identified in the 2019 study on the heel (Morris et al., 2019) are most likely to have significant effects on bone mineral density based on the eQTLs they contain. This computational approach is known as a transcriptome wide association study or TWAS. Next, Al-Barghouthi et al. performed eQTL colocalization, a statistical technique that can determine whether an eQTL variant found in a specific region of the genome is driving the GWAS signal in that region. This approach allows researchers to identify specific sequences or genes that are likely responsible for a trait. Combining these two approaches, the team identified 512 genes that could potentially cause osteoporosis.

To confirm that their method was capturing genes regulating bone mineral density, Al-Barghouthi et al. asked whether the genes they had identified were enriched for known bone genes. They found that about 13% of the genes they had captured were known bone genes, over what would be expected by chance. Identified genes were also enriched for ontologies associated with bone (i.e. ossification, skeletal system development, and osteoblast differentiation). Additionally, Al-Barghouthi et al. attempted to validate their data using information on bone mineral density from knockout mice available through the International Mouse Phenotype Consortium (Dickinson et al., 2016; Swan et al., 2020). This consortium had available knockouts for 142 out of the 512 genes. Of these, 64 mutants led to alteration of whole-body bone mineral density. An exciting finding was that a large percentage (76.5%) of genes that cause changes in bone mineral density in mice knockouts did not belong to a ‘known bone gene’, which gives us a hint about the complexity of the biological mechanisms that regulate bone mineral density.

Finally, Al-Barghouthi et al. tightened their analysis to capture genes with the strongest evidence for causality. PPP6R3 was the second top gene identified and the first without functional evidence for bone mineral density. Surprisingly, the associated locus that PPP6R3 belongs to is very well known, as it harbors a well-studied bone gene called LRP5. The question therefore arises: is PPP6R3 a new gene in bone mineral density regulation, or is the GWAS signal associated with LRP5? To answer this question, Al-Barghouthi et al. generated mice knockouts for *Ppp6r3*, the mouse version of the gene, and then measured their bone mineral density at the femur and the lumbar spine. They found that in addition to showing lower bone mineral density at the spine, the *Ppp6r3* knockout mice demonstrated deterioration of bone microarchitecture and increased bone turnover, which is commonly observed in models of osteoporosis.

One limitation of the work of Al-Barghouthi et al. is that the GTEx project does not contain expression data from bone tissues. However, due to the complex nature of bone mineral density, new genes can still be identified using their approach. Of course, questions still remain regarding the direct or indirect effect of genes on bone mineral density. For example, how does PPP6R3 regulate osteoblast or osteoclast activity? Another important finding of the latest work is that many of the genes potentially causing osteoporosis are in gene-rich regions of the genome. Indeed, as PPP6R3 and LRP5 demonstrate, a single associated genomic region may harbor more than one causative gene.

While a follow up from this work is necessary to test the causality of other identified genes using animal models, it is equally important to explore if and how PPP6R3 contributes to fracture risk and osteoporosis. In the future, approaches like the one used by Al-Barghouthi et al. will allow researchers to slowly learn more about the genetic causes of osteoporosis, which could lead to better treatments.

## References

[bib1] Al-Barghouthi BM, Rosenow WT, Du K-P, Heo J, Maynard R, Mesner L, Calabrese G, Nakasone A, Senwar B, Gerstenfeld L, Larner J, Ferguson V, Ackert-Bicknell C, Morgan E, Brautigan D, Farber CR (2022). Transcriptome-wide association study and eQTL colocalization identify potentially causal genes responsible for human bone mineral density GWAS associations. eLife.

[bib2] Dickinson ME, Flenniken AM, Ji X, Teboul L, Wong MD, White JK, Meehan TF, Weninger WJ, Westerberg H, Adissu H, Baker CN, Bower L, Brown JM, Caddle LB, Chiani F, Clary D, Cleak J, Daly MJ, Denegre JM, Doe B, Dolan ME, Edie SM, Fuchs H, Gailus-Durner V, Galli A, Gambadoro A, Gallegos J, Guo S, Horner NR, Hsu CW, Johnson SJ, Kalaga S, Keith LC, Lanoue L, Lawson TN, Lek M, Mark M, Marschall S, Mason J, McElwee ML, Newbigging S, Nutter LMJ, Peterson KA, Ramirez-Solis R, Rowland DJ, Ryder E, Samocha KE, Seavitt JR, Selloum M, Szoke-Kovacs Z, Tamura M, Trainor AG, Tudose I, Wakana S, Warren J, Wendling O, West DB, Wong L, Yoshiki A, MacArthur DG, Tocchini-Valentini GP, Gao X, Flicek P, Bradley A, Skarnes WC, Justice MJ, Parkinson HE, Moore M, Wells S, Braun RE, Svenson KL, de Angelis MH, Herault Y, Mohun T, Mallon AM, Henkelman RM, Brown SDM, Adams DJ, Lloyd KCK, McKerlie C, Beaudet AL, Bućan M, Murray SA, International Mouse Phenotyping Consortium, Jackson Laboratory, Infrastructure Nationale PHENOMIN, Institut Clinique de la Souris (ICS), Charles River Laboratories, MRC Harwell, Toronto Centre for Phenogenomics, Wellcome Trust Sanger Institute, RIKEN BioResource Center (2016). High-throughput discovery of novel developmental phenotypes. Nature.

[bib3] GTEx Consortium (2013). The genotype-tissue expression (GTEx) project. Nature Genetics.

[bib4] Kague E, Medina-Gomez C, Boyadjiev SA, Rivadeneira F (2022). The genetic overlap between osteoporosis and craniosynostosis. Frontiers in Endocrinology.

[bib5] Morris JA, Kemp JP, Youlten SE, Laurent L, Logan JG, Chai RC, Vulpescu NA, Forgetta V, Kleinman A, Mohanty ST, Sergio CM, Quinn J, Nguyen-Yamamoto L, Luco A-L, Vijay J, Simon M-M, Pramatarova A, Medina-Gomez C, Trajanoska K, Ghirardello EJ, Butterfield NC, Curry KF, Leitch VD, Sparkes PC, Adoum A-T, Mannan NS, Komla-Ebri DSK, Pollard AS, Dewhurst HF, Hassall TAD, Beltejar M-JG, Adams DJ, Vaillancourt SM, Kaptoge S, Baldock P, Cooper C, Reeve J, Ntzani EE, Evangelou E, Ohlsson C, Karasik D, Rivadeneira F, Kiel DP, Tobias JH, Gregson CL, Harvey NC, Grundberg E, Goltzman D, Adams DJ, Lelliott CJ, Hinds DA, Ackert-Bicknell CL, Hsu Y-H, Maurano MT, Croucher PI, Williams GR, Bassett JHD, Evans DM, Richards JB, 23andMe Research Team (2019). An atlas of genetic influences on osteoporosis in humans and mice. Nature Genetics.

[bib6] Ralston SH, Uitterlinden AG (2010). Genetics of osteoporosis. Endocrine Reviews.

[bib7] Salari N, Ghasemi H, Mohammadi L, Behzadi MH, Rabieenia E, Shohaimi S, Mohammadi M (2021). The global prevalence of osteoporosis in the world: a comprehensive systematic review and meta-analysis. Journal of Orthopaedic Surgery and Research.

[bib8] Swan AL, Schütt C, Rozman J, Del Mar Muñiz Moreno M, Brandmaier S, Simon M, Leuchtenberger S, Griffiths M, Brommage R, Keskivali-Bond P, Grallert H, Werner T, Teperino R, Becker L, Miller G, Moshiri A, Seavitt JR, Cissell DD, Meehan TF, Acar EF, Lelliott CJ, Flenniken AM, Champy MF, Sorg T, Ayadi A, Braun RE, Cater H, Dickinson ME, Flicek P, Gallegos J, Ghirardello EJ, Heaney JD, Jacquot S, Lally C, Logan JG, Teboul L, Mason J, Spielmann N, McKerlie C, Murray SA, Nutter LMJ, Odfalk KF, Parkinson H, Prochazka J, Reynolds CL, Selloum M, Spoutil F, Svenson KL, Vales TS, Wells SE, White JK, Sedlacek R, Wurst W, Lloyd KCK, Croucher PI, Fuchs H, Williams GR, Bassett JHD, Gailus-Durner V, Herault Y, Mallon AM, Brown SDM, Mayer-Kuckuk P, Hrabe de Angelis M, IMPC Consortium (2020). Mouse mutant phenotyping at scale reveals novel genes controlling bone mineral density. PLOS Genetics.

[bib9] Timpson NJ, Greenwood CMT, Soranzo N, Lawson DJ, Richards JB (2018). Genetic architecture: the shape of the genetic contribution to human traits and disease. Nature Reviews Genetics.

[bib10] Trajanoska K, Morris JA, Oei L, Zheng HF, Evans DM, Kiel DP, Ohlsson C, Richards JB, Rivadeneira F, GEFOS/GENOMOS consortium and the 23andMe research team (2018). Assessment of the genetic and clinical determinants of fracture risk: genome wide association and mendelian randomisation study. BMJ.

